# The Story of the Hospitals

**Published:** 1886-11-13

**Authors:** 


### The Story of the Hospitals.

(*Continued from page 56.*)

Howard at the "London."

Before I describe the work, carried out with such marvellous precision and organisation as it is to-day, I will pause while I ask my readers to take with me a peep into the past. For this purpose we will take as our "guide, philosopher, and friend" that distinguished philanthropist, John Howard. Nearly a century ago (15th September, 1788) this large-hearted man paid a visit to the London Hospital, and he has left on record in his "Brief Account of the Hospitals for the Sick in this Great Metropolis," his own impressions of this institution as it existed in his day. Comparisons are proverbially odious, but, in the present instance, we shall happily find the "odium" resting on the past, and not on the present, administration of the hospital. Mighty indeed have been the changes for the better.

The "London," 1788.

The philanthropist begins by telling us that this "spacious building" is intended for the reception and relief of sick and wounded seamen, etc., and consists, he says, of eighteen wards; but, he adds, "seven only are occupied, some having been shut up two or three years." The wards to-day are 78 in number, and none are shut! When Howard visited it, he found 120 patients there; now there is something like an average of 700 beds in constant occupation. The ventilation of the wards was of a very primitive character, consisting of "square apertures" over the doors of the wards. The passages, the philanthropist describes as "about eight feet wide, and dark." There were, he tells us, no cisterns for water, and the sanitary arrangements were exceedingly bad. Medical and surgical patients were mixed indiscriminately together. "I could wish," writes Howard, "that there were two wards appropriated to Jew patients, as they must almost starve on their scanty allowance of bread and beer, with only twopence-halfpenny a day." Howard's wish did not find a practical realisation until 1840, when the centenary festival was held. The large addition which was then made to the hospital enabled the governors to appropriate a portion of the building to the exclusive use of Jewish patients.

Howard on the "Diet."

The philanthropist writes: "The patients' diet I disapprove of, as their *common diet* is 8 oz. of meat *every* day for dinner, and for supper, broth six days in the week. *No vegetables*, and only 12 oz. of bread a day. The *middle diet* is 4 oz. of meat every day for dinner; and for supper, a pint of broth or panado (bread boiled in water and sweetened). *No vegetables*, and only 8 oz. of bread. The breakfast for every day of those patients that are on *common diet*, is one pint of milk pottage, or water-gruel; those on *middle diet*, one pint of panado or water-gruel. The drink of the former is three pints of beer in summer and one quart in winter; of the latter, one pint of beer every day." Howard's disapproval of the diet table will be better understood when we remember that he was an ultra-vegetarian. Eight ounces of meat per diem for a patient on common diet, and four to those on middle diet, must have appeared a sin in the eyes of this staunch believer in the doctrine that vegetable substances are the solids which Dame Nature has decreed that man shall eat. The diet at the London Hospital to-day differs much from the table given by Howard. Patients on full diet have still their 12 oz. of bread, but the meat has been reduced to 6 oz., and 8 oz. of bread have been added, while those on middle diet have similar allowances of bread and potatoes and 4 oz. of meat. The consumption of malt liquor at the London Hospital in Howard's time must have been, relatively speaking, enormously greater than it is to-day. The use of alcoholic beverages has, in recent years, gradually and steadily diminished. In place of the beer in Howard's table, milk is everywhere given, unless porter is specially ordered by the physician, surgeon, or his representative.

Order Everywhere.

The London Hospital, as it exists to-day, may certainly be regarded as one of the sights of London. No matter to what part of the building we may direct our steps, we are certain to be impressed by the gigantic character of the work, the order, the promptitude, the regulation, which reign everywhere supreme. Everybody appears to set about his or her duty with a business-like yet quiet energy. There is little that escapes the keen glance of the house-governor, who appears to perceive in an instant the smallest thing which is not quite *en règle*. The same remark applies also to the matron in connection with the nursing staff. But the methodical and systematic routine which we find at the London Hospital to-day did not always exist. In the time of the former house-governor—an old military man—things were very different from what they are now. Mr. Nixon has seen a period of forty years' service at the London, during the last twenty of which he has discharged the onerous duties of house-governor, and he has applied himself zealously to the correction of abuses, with a view to a more efficient economy in the management of the hospital.

The Out-patients' Department.

The remodelling of the out-patients' department is a work on which Mr. Nixon has brought his great experience to bear with a highly successful result. Abuses, of course, exist at all hospitals in relief of this kind, and the only thing which it is possible to do is to reduce them to a minimum. With this object in view, Mr. Nixon determined, some four or five years ago, to tackle this perplexing question. The increasing population of the East End, dependent mainly on the resources of this great central charity, necessitated of itself some radical change in the free out-patient system. It was evident also that many of the applicants—more particularly in the special departments—belonged to classes who ought not to obtain gratuitous advice and treatment. It was therefore determined to draft such a scheme of reform as would eliminate unsuitable cases, but without injury to the necessitous poor. On 1st January, 1884, the revised regulations came into operation. In order to check the abuse of charity, a waiting-hall inspector was appointed to ascertain whether patients, presenting governors' letters, were able to pay the fees of consulting physicians or surgeons, or (not being urgent cases) should not be referred to local practitioners, etc. While, under the new system, no person, whatever his social position may be, is refused a first attendance, the admission of a patient, and his subsequent continuance under treatment, are practically under the control of the medical or surgical officer, if deemed necessary for urgency or clinical purposes. The practical result of the first year's trial of the new system was a reduction in the number of out-patients—chiefly in the skin, ear, and eye departments—to the extent of nearly 7,000. As each patient in 1884 attended on an average $3\frac{3}{4}$ times, this reduction means something like 26,000 fewer attendances in the year. The house-governor has gone very closely into this question, and he has convinced himself that, while the waiting-halls have been relieved of much of their over-pressure, from which they formerly suffered, no injury has been done to the necessitous poor.

*(To be continued.)*

